# Spatiotemporal distribution, abundance, and host interactions of two invasive vectors of arboviruses, *Aedes albopictus* and *Aedes japonicus*, in Pennsylvania, USA

**DOI:** 10.1186/s13071-022-05151-8

**Published:** 2022-01-24

**Authors:** Eliza A. H. Little, Michael L. Hutchinson, Keith J. Price, Alyssa Marini, John J. Shepard, Goudarz Molaei

**Affiliations:** 1 Department of Entomology, The Connecticut Agricultural Experiment Station, 123 Huntington Street, New Haven, CT 06511 USA; 2 Center for Vector Biology and Zoonotic Diseases and Northeast Regional Center for Excellence in Vector-Borne Diseases, The Connecticut Agricultural Experiment Station, 123 Huntington Street, New Haven, CT 06511 USA; 3 Present Address: Pennsylvania Department of Agriculture, 2301 North Cameron Street, Harrisburg, PA 17110 USA; 4 Pennsylvania Department of Environmental Protection, 400 Market Street, Harrisburg, PA 17101 USA; 5 Department of Environmental Sciences, The Connecticut Agricultural Experiment Station, 123 Huntington Street, New Haven, CT 06511 USA; 6 Department of Epidemiology of Microbial Diseases, Yale School of Public Health, 60 College Street, New Haven, CT 06510 USA

## Abstract

**Background:**

*Aedes albopictus* and *Aedes japonicus*, two invasive mosquito species in the United States, are implicated in the transmission of arboviruses. Studies have shown interactions of these two mosquito species with a variety of vertebrate hosts; however, regional differences exist and may influence their contribution to arbovirus transmission.

**Methods:**

We investigated the distribution, abundance, host interactions, and West Nile virus infection prevalence of *Ae. albopictus* and *Ae. japonicus* by examining Pennsylvania mosquito and arbovirus surveillance data for the period between 2010 and 2018. Mosquitoes were primarily collected using gravid traps and BG-Sentinel traps, and sources of blood meals were determined by analyzing mitochondrial cytochrome b gene sequences amplified in PCR assays.

**Results:**

A total of 10,878,727 female mosquitoes representing 51 species were collected in Pennsylvania over the 9-year study period, with *Ae. albopictus* and *Ae. japonicus* representing 4.06% and 3.02% of all collected mosquitoes, respectively. *Aedes albopictus* was distributed in 39 counties and *Ae. japonicus* in all 67 counties, and the abundance of these species increased between 2010 and 2018. Models suggested an increase in the spatial extent of *Ae. albopictus* during the study period, while that of *Ae. japonicus* remained unchanged. We found a differential association between the abundance of the two mosquito species and environmental conditions, percent development, and median household income. Of 110 *Ae. albopictus* and 97 *Ae. japonicus* blood meals successfully identified to species level, 98% and 100% were derived from mammalian hosts, respectively. Among 12 mammalian species, domestic cats, humans, and white-tailed deer served as the most frequent hosts for the two mosquito species. A limited number of *Ae. albopictus* acquired blood meals from avian hosts solely or in mixed blood meals. West Nile virus was detected in 31 pools (*n* = 3582 total number of pools) of *Ae. albopictus* and 12 pools (*n* = 977 total pools) of *Ae. japonicus*.

**Conclusions:**

Extensive distribution, high abundance, and frequent interactions with mammalian hosts suggest potential involvement of *Ae. albopictus* and *Ae. japonicus* in the transmission of human arboviruses including Cache Valley, Jamestown Canyon, La Crosse, dengue, chikungunya, and Zika should any of these viruses become prevalent in Pennsylvania. Limited interaction with avian hosts suggests that *Ae. albopictus* might occasionally be involved in transmission of arboviruses such as West Nile in the region.

**Graphical Abstract:**

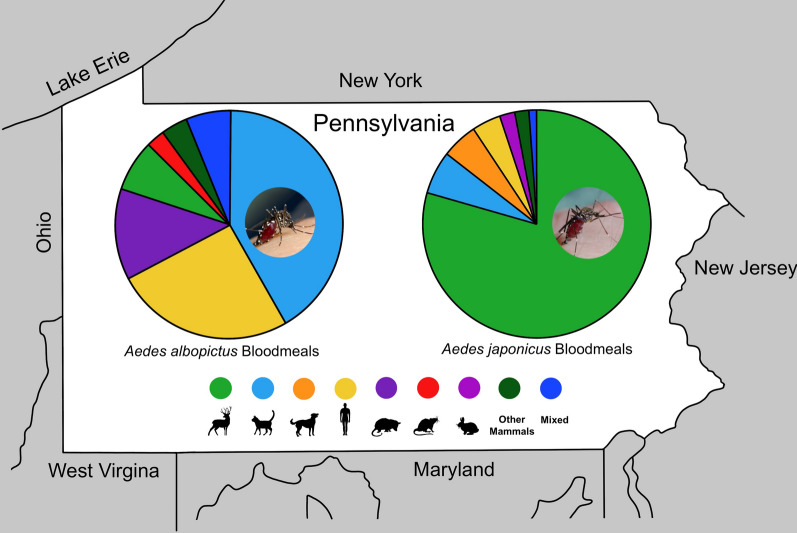

**Supplementary Information:**

The online version contains supplementary material available at 10.1186/s13071-022-05151-8.

## Background

The mosquito genus *Aedes* has garnered international attention in recent years after the emergence and rapid spread of Zika virus (ZIKV) infections in Central and South America, the Caribbean, and the state of Florida in the United States [1, 2]. Native to Asia, *Aedes albopictus* was first introduced into the United States in Texas in 1985 [3] and has since spread to 38 states [4, 5]. Also introduced from Asia, *Aedes japonicus* was first reported in the United States in Connecticut in 1997 [6, 7], New York and New Jersey in 1998 [8], and Pennsylvania in 1999 [9]. *Aedes japonicus* is now found in 33 states, [10, 11]. Both *Ae. albopictus* and *Ae. japonicus* are container-inhabiting mosquitoes that take advantage of natural and artificial containers and thrive in peridomestic environments [12]. The spread of both *Aedes* species is inextricably linked to these artificial containers (e.g., tires) transported across infrastructure (i.e., highways) [3, 13]. Their successful invasion is due in large part to their adaptability to a wide range of environmental conditions in temperate climates and human environments.

Not only have *Ae. albopictus* and *Ae. japonicus* successfully invaded temperate North America, but there is evidence to suggest that under certain conditions they may outcompete native mosquito species including *Aedes triseriatus* [14, 15]. It is also suggested that this species is outcompeting *Aedes atropalpus* in some areas of the United States due to shorter larval development periods [16]. Bearing highly adaptive traits and exhibiting competitive advantages over native mosquito species, *Ae. albopictus* and *Ae. japonicus* may alter mosquito biodiversity and indirectly influence the epidemiology of mosquito-borne diseases [10]. Co-occurrence of these two species has also affected interspecific competition, with *Ae. albopictus* generally outcompeting *Ae. japonicus* in larval habitats [17]. Although *Ae. albopictus* has been shown to be superior to *Ae. japonicus* in competing for food resources in larval habitats in the United States (particularly in artificial container habitats), higher overwintering survival and earlier hatching means that *Ae. japonicus* is able to exploit larval habitats before *Ae. albopictus* [15, 18]. Field observations suggest that *Ae. albopictus* are more abundant in urban and suburban areas while *Ae. japonicus* are more common in rural areas [12]. This distinction in habitat niche may be due to differences in temperature tolerance. *Aedes japonicus* is a temperate mosquito, primarily distributed in cooler latitudes in its native and invaded ranges [10]. Hot, dry summer conditions mediated by climate change and urban heat islands may negatively impact *Ae. japonicus* distribution, especially in highly urbanized areas, whereas these conditions are more favorable to increased populations of *Ae. albopictus* [19].

Mosquito–host interactions are important for assessing vectorial capacity in *Aedes* populations and estimating the risk of arbovirus transmission. Host interaction studies show that *Ae. albopictus* obtains blood meals predominantly from a variety of mammalian hosts including humans, domestic cats, brown rats, dogs, opossum, rabbits, deer, and squirrels. Human-derived blood meals have been identified in 50–100% of *Ae. albopictus* across many studies [20–27]. However, opportunistic blood-feeding of this mosquito species from a wide variety of mammalian hosts has been reported in other investigations [28–30]. *Aedes albopictus* has also been reported to obtain blood meals from avian, reptilian, and amphibian hosts [21, 27, 28, 31–35]. Collectively, these studies indicate that *Ae. albopictus* interacts with a variety of host species and potentially contributes to epizootic-epidemic transmission of arboviruses in different regions.

Previous studies have demonstrated that *Ae. japonicus* is associated exclusively with mammalian hosts in blood-feeding [21, 29, 36–41] in North America. Multiple studies in the northeastern United States have found that white-tailed deer, the most abundant large mammal in the region, represent the majority of blood meals identified from *Ae. japonicus* [36–39]. But other mammalian hosts have also been identified including the domestic cat [29, 40], brown rat [29], opossum [38], cow [41], chipmunk [37], and horse [36, 38]. Opportunistic blood-feeding suggests that *Ae. japonicus* may be an important vector for arboviruses involving small and medium-sized mammalian hosts [29, 42]. The potential for *Ae. japonicus* to act as a “bridge vector” for West Nile virus (WNV) cannot be entirely discounted, because it has been shown to feed on both humans [29, 37, 38, 40] and birds in the laboratory [43] and in the field [44], albeit at lower frequencies.

*Aedes albopictus* and *Ae. japonicus* are vectors for viral pathogens causing diseases in animals and humans. Multiple arboviruses have been isolated from field-collected *Ae. albopictus* including Cache Valley virus (CVV), eastern equine encephalitis virus (EEEV), Jamestown Canyon virus (JCV), La Crosse virus (LACV), and WNV [45, 46]. Local transmission of other arboviruses including dengue (DENV), chikungunya (CHIKV), and ZIKV by established populations of *Ae. albopictus* has occurred in temperate areas [47–52].

*Aedes japonicus* in its native range has been implicated in Japanese encephalitis virus (JEV) outbreaks [53]. In laboratory studies, *Ae. japonicus* has been shown to be a competent vector of LACV [54], WNV [55], St. Louis encephalitis virus (SLEV) [56], EEEV [57], DENV, CHIKV [58], and Rift Valley fever virus (RVFV) [59]. In the United States, WNV [60–62], LACV [40, 42], and CVV [63] have been isolated from field-collected *Ae. japonicus*.

Urban landscapes impact the spatial variability of mosquito abundance [35], community composition [12], mosquito–host interactions [25, 30, 33, 34], and infection rates [64, 65]. Because of their vector competence, close association with and blood-feeding on humans, *Ae. albopictus* and *Ae. japonicus* are considered vectors of public health importance. Thus, a better understanding of the impact of urban landscapes on mosquito abundance, blood-feeding, and infection status of *Ae. albopictus* and *Ae. japonicus* is vital for mitigating the risk of human infection with arboviruses. WNV is of particular concern as it is the most common arbovirus in the United States and is the only arbovirus known to cause significant human disease in Pennsylvania. In Pennsylvania in 2018, the incidence of WNV neuroinvasive disease (0.74 per 100,000) was > 35% higher than the median national incidence and was highest among New England and mid-Atlantic states [66]. In this study, our objectives were to (1) explore spatial and temporal changes in the distribution and abundance of *Ae. albopictus* and *Ae. japonicus*, (2) assess the influence of urban landscapes on their abundance and blood-feeding patterns, and (3) investigate *Ae. albopictus* and *Ae. japonicus* infection status with WNV in Pennsylvania between 2010 and 2018.

## Methods

### Mosquito collection

**Fig. 1:**
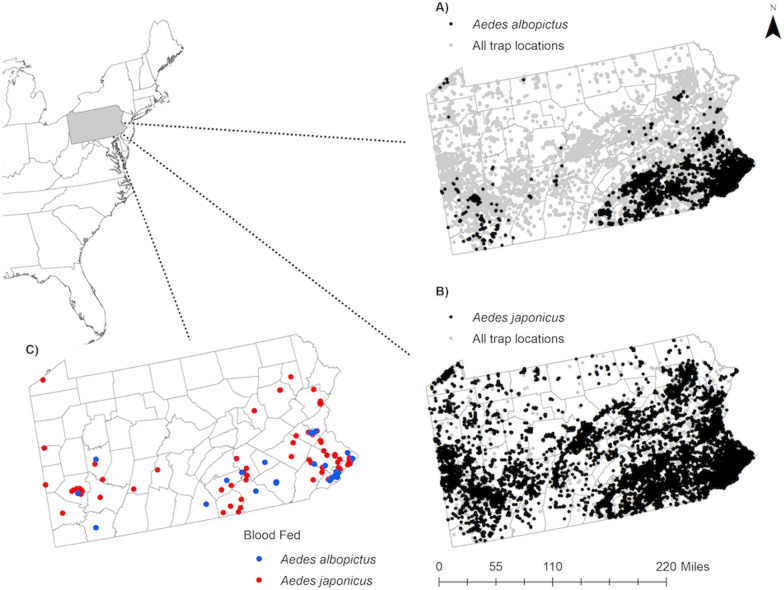
Location of Pennsylvania in the northeastern United States and **a** traps containing *Ae. albopictus*, **b** traps containing *Ae. japonicus*, and **c** locations of blood-fed *Ae. albopictus* and *Ae. japonicus*

Mosquitoes were collected in Pennsylvania from 2010 to 2018 as part of a statewide arbovirus surveillance program (Fig. 1). Most adult collections were made from April through October, with some occurring outside that time frame, including collections made from winter hibernacula. Surveillance was conducted in all 67 counties from over 19,000 unique collection sites. The surveillance program had a heavy emphasis on the detection of WNV in *Culex* mosquitoes in urban and suburban environs. Therefore, mosquito collections were largely, but not exclusively, focused near human population centers. Surveillance sites included wastewater treatment facilities, manure pits on farms, stormwater retention and detention basins, green spaces, wetlands, residential properties, salvage yards, tire recycling facilities, and other locations.

Trapping methodologies included the use of gravid traps baited most frequently with a hay/lactalbumin infusion (2800 Series, BioQuip products, Rancho Dominguez, CA, USA), Centers for Disease Control and Prevention (CDC) miniature light traps baited with carbon dioxide (John W. Hock Co., Gainesville, FL, USA), Biogents BG-Sentinel traps baited with BG-Lure and carbon dioxide (Biogents, Regensburg, Germany), aspiration with handheld aspirators (John W. Hock Co., Gainesville, FL, USA), Fay-Prince omnidirectional traps baited with carbon dioxide (John W. Hock Co., Gainesville, FL, USA), Mosquito Magnet traps baited with carbon dioxide and 1-octen-3-ol (American Biophysics Corp., Kingstown, RI, USA), resting boxes (constructed by Department of Environmental Protection staff), and Zumba traps baited with carbon dioxide (ISCA Technologies, Riverside, CA, USA). Traps were typically set overnight and collected the following morning. Biogents BG-Sentinel traps were frequently run for 24 h to increase collection success for *Ae. albopictus*, which can be highly active during the day. In some cases, particularly with the Mosquito Magnet, traps were allowed to run for multiple days before collection. Mosquitoes collected from gravid and Biogents BG-Sentinel traps represented 94.6% of all collected mosquitoes. Mosquitoes were either shipped to the Department of Environmental Protection laboratory overnight on dry ice or delivered to the lab alive and euthanized in a −80 °C freezer.

### Mosquito processing

Mosquitoes were morphologically identified, sorted, enumerated, and pooled (vector spp.) on a chill table with the aid of a Leica MZ7.5 stereomicroscope (Leica Microsystems, Wetzlar, Germany) using descriptive keys [67–69]. Specimens were identified to the lowest practical taxonomic level, typically species level, but often grouped by genus or species groups (e.g., *Culex pipiens/restuans*) for purposes of pooling specimens to maximize virus testing efficiency. Specimens retained for blood meal analysis were placed in 1.5 ml microtubes and labeled accordingly. If multiple engorged specimens were collected from a single sample, they were retained communally in the same tube unless the abdomens were visibly damaged, in which case those specimens were placed in tubes singly to avoid cross-contamination. The tubes were then placed in a − 80 °C freezer until further processing. *Aedes japonicus* with visible blood in their abdomens were retained from 2010 to 2015, while *Ae. albopictus* were retained in 2018.

### Pathogen testing (virus isolation and identification)

Specimens retained for the intent of virus testing were pooled into 11 ml polypropylene tubes (Sarstedt, Nümbrecht, Germany) by species (or other relevant taxon) of typically up to 100, but occasionally up to 200, specimens per tube and linked with their associated collection data. Mosquito pools were homogenized in tubes containing four 4.5 mm-diameter copper-coated steel beads and 1–2.5 ml BA-1 diluent [70]. Tubes were placed in a multi-tube vortexer (Fisher Scientific, Waltham, MA, USA) for 60 s and the homogenate centrifuged (Allegra 25R centrifuge, Beckman Coulter, Inc., Brea, CA, USA) at 3571×*g* for 10 min at 4 °C. Subsamples of mosquito pool homogenates (220 µl) were then transferred to a 96-well S-block containing 280 µl lysis buffer AL/carrier RNA mix and Qiagen protease and incubated at 56 °C for 10 min. All assays included no template controls [Buffer AVE, RNase-free water with 0.04% NaN_3_ (Qiagen, Hilden, Germany)], negative control (real-time reverse transcription polymerase chain reaction) [RT-PCR]-negative mosquito pool homogenate), and positive control (fivefold dilution of virus-infected tissue culture). Nucleic acids were purified using the QIAamp Virus BioRobot MDx Kit (Qiagen) on the Qiagen BioRobot Universal System following manufacturer-recommend procedures and eluted in 75 µl AVE buffer (Qiagen).

RT-PCR assays to detect WNV in pools targeted the 3′ untranslated region [70], and SLEV and LACV assays targeted the NS5 gene and M segment of the viral genome using primers and probes, respectively [71, 72]. A second primer/probe set targeting the envelop (E) gene was used as necessary for confirmatory tests [70]. Probes were labeled with 5′-6-carboxyfluorescein (FAM) reporter dye and 3′-6-carboxytetramethylrhodamine (TAMRA) quencher (Thermo Fisher Scientific, Waltham, MA, USA).

Reaction mixtures contained 0.80 µM of each primer, 0.20 µM probe, 8 µl 2× qScript One-Step master mix, Low ROX (Quantabio, Beverly, MA, USA), 0.32 μl qScript One-Step reverse transcriptase, 7.2 μl nuclease-free water, and 4 µl RNA template in 20 μl total reaction volume. RT-PCR was performed using the 7500 Real-Time PCR System (Applied Biosystems, Foster City, CA, USA) with the following cycling conditions: 48 °C for 10 min followed by 95 °C for 5 min and 40 cycles of 95 °C for 15 s and 60 °C for 1 min. Samples were considered positive for cycle threshold (Ct) values ≤ 38, and samples with low viral loads, i.e., Ct > 38, were confirmed by targeting the E gene.

### Blood meal identification in engorged *Ae. japonicus* and *Ae. albopictus* mosquitoes

To identify the sources of blood meals in engorged mosquitoes, abdomens were individually dissected on microscope slides using sterile razor blades with the aid of a stereomicroscope. Extraction of genomic DNA from the mosquito abdomens was performed using the DNeasy Blood & Tissue Kit (Qiagen, Valencia, CA, USA) or DNAzol BD (Molecular Research Center, Cincinnati, OH, USA) according to the manufacturer’s suggested protocols with modifications described elsewhere [30, 37, 73, 74]. PCR assays on extracted DNA were conducted using primers based on the vertebrate mitochondrial cytochrome *b* gene [73, 75, 76] and Taq PCR Core Kit (Qiagen). DNA samples isolated from the blood of several vertebrate species were used in PCR reactions as positive control [74]. UltraPure DNase/RNase-free-molecular biology-grade distilled water (Invitrogen by Life Technologies, Grand Island, NY, USA) was used as negative control. Detailed PCR protocols including reaction mixtures and thermal cycling conditions have been described elsewhere [73, 76]. PCR-amplified products were purified using the QIAquick PCR Purification Kit (Qiagen) and sequenced in forward and reverse directions using Sanger sequencing on a 3730xl DNA Analyzer (Applied Biosystems, Foster City, CA, USA) at the Keck Sequencing Facility (Yale University, New Haven, CT, USA). ChromasPro version 1.7.5 (Technelysium Pty Ltd., Tewantin, Australia) was used to annotate the sequences. Sequences were compared to the sequences in the NCBI GenBank (https://blast.ncbi.nlm.nih.gov/Blast.cgi?PROGRAM=blastn&PAGE_TYPE=BlastSearch&LINK_LOC=blasthome) using the BLASTn search tool. A positive identification was made when > 97% identity was attained between the query and subject sequence.

### Statistical analysis

Maximum likelihood estimation (MLE) is considered the most appropriate estimate of infection rate when pool size varies [77]. To estimate annual infection rates across Pennsylvania, we calculated the MLE as previously described [78] for all mosquito species that had at least one positive pool. Further, we calculated infection rates using MLE per 1000 mosquitoes by location for *Ae. albopictus* and *Ae. japonicus*.

**Fig. 2:**
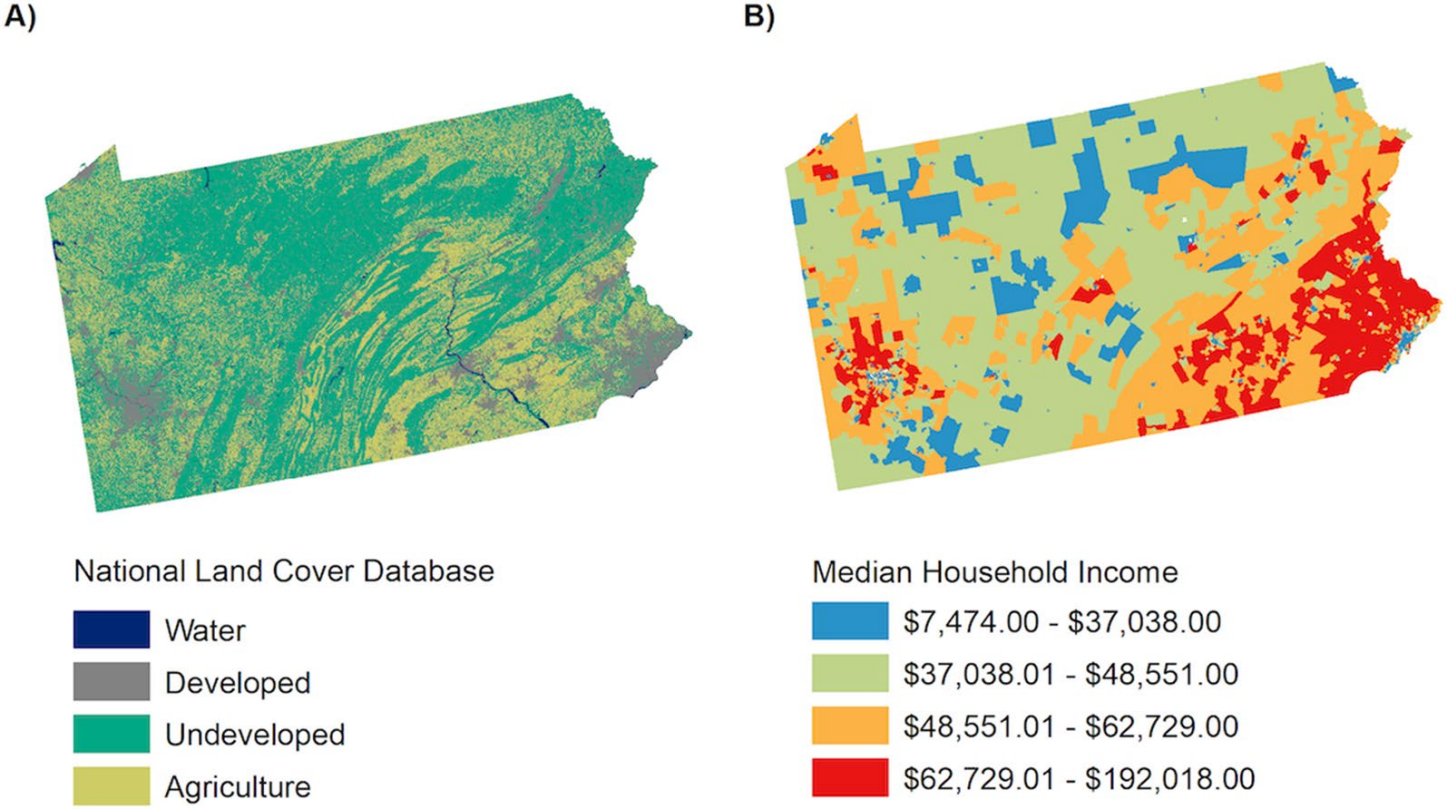
Explanatory variables percent development derived from the National Land Cover Database (**a**) and median household income (**b**) in Pennsylvania

To explore relationships between urban landscapes and *Ae. albopictus* and *Ae. japonicus* abundance*,* we accessed spatially explicit, freely available data on development (DEV) and median household income (MHI). The 2016 National Land Cover Database classification was simplified into four classes characterizing water, developed, undeveloped, and agricultural land cover [30]. In ArcGIS, we calculated the proportion of developed land within a radius of 200 m of each trap location to measure the influence of urban landscapes on *Aedes* mosquitoes (Fig. 2). For each census tract in Pennsylvania, we accessed the United States Census 2010 estimates of MHI (US Census Bureau 2010, Table S1903). In ArcGIS, we extracted this estimate of MHI at each trap location (Fig. 2). We standardized the environmental conditions, the DEV and MHI, across all trap locations by subtracting the mean and dividing by the standard deviation.

We used contingency tables to compare abundance and blood meals across environmental conditions split at the mean, and generalized linear mixed effects models (GLMM) to evaluate how the urban landscape, DEV and MHI, influences *Ae. albopictus* and *Ae. japonicus* abundance (family = Poisson), blood-feeding (family = binomial), and WNV infection rates (family = Poisson). We used mixed-model regression to accommodate the temporal structure of the data, with year as a random effect. All statistical analyses were completed using R Statistical Software version 3.6.2 [79] and maps were created in ArcGIS version 10.8 (Esri, Redlands, CA, USA).

## Results

Across all trap types, a total of 10,878,727 female mosquitoes were collected between 2010 and 2018. The most frequently collected species were *Cx. restuans* (42.58%; *n* = 4,631,831) and *Cx. pipiens* (25.50%; *n* = 2,774,163), together with those identified as either *Culex* species (12.48%; *n* = 1,358,060), comprising 80.56% (*n* = 8,764,054) of the total collection (Table 1). *Aedes albopictus* represented 4.06% (*n* = 441,542) and *Ae. japonicus* represented 3.02% (*n* = 328,438) of all mosquitoes collected between 2010 and 2018 (Table 1). Gravid traps were by far the most common trap types used, representing 85% of all traps, followed by BG-Sentinel traps (9.6%).

**Table 1:** Number of adult female mosquitoes collected from trap locations in Pennsylvania between 2010 and 2018

Species	Total	Percent (%)
*Culex restuans*	4,631,831	42.58
*Culex pipiens*	2,774,163	25.50
*Culex pipiens/restuans*	1,358,060	12.48
*Aedes albopictus*	441,542	4.06
*Aedes japonicus*	328,438	3.02
*Aedes trivittatus*	282,458	2.60
*Aedes vexans*	243,617	2.24
*Culex salinarius*	145,814	1.34
*Psorophora ferox*	105,443	0.97
*Aedes triseriatus*	62,858	0.58
*Coquillettidia perturbans*	57,787	0.53
*Aedes canadensis*	56,156	0.52
*Aedes sticticus/trivittatus*	54,844	0.50
*Anopheles punctipennis*	47,689	0.44
*Aedes sticticus*	45,233	0.42
*Culex erraticus*	36,995	0.34
*Anopheles quadrimaculatus*	21,224	0.20
*Aedes stimulans*	9551	0.09
*Culex territans*	9484	0.09
*Psorophora columbiae*	5547	0.05
*Aedes cinereus*	3061	0.03
*Aedes dorsalis*	2017	0.02
*Uranotaenia sapphirina*	1343	0.01
*Anopheles barberi*	1240	0.01
*Aedes cantator*	1198	0.01
*Culiseta minnesotae*	1123	0.01
*Psorophora ciliata*	937	0.01
*Anopheles walkeri*	755	0.01
*Aedes atropalpus*	675	0.01
Other spp.	147,644	1.36
Total	10,878,727	100

### Temporal and spatial changes in the abundance of *Ae. albopictus* and *Ae. japonicus*

**Fig. 3:**
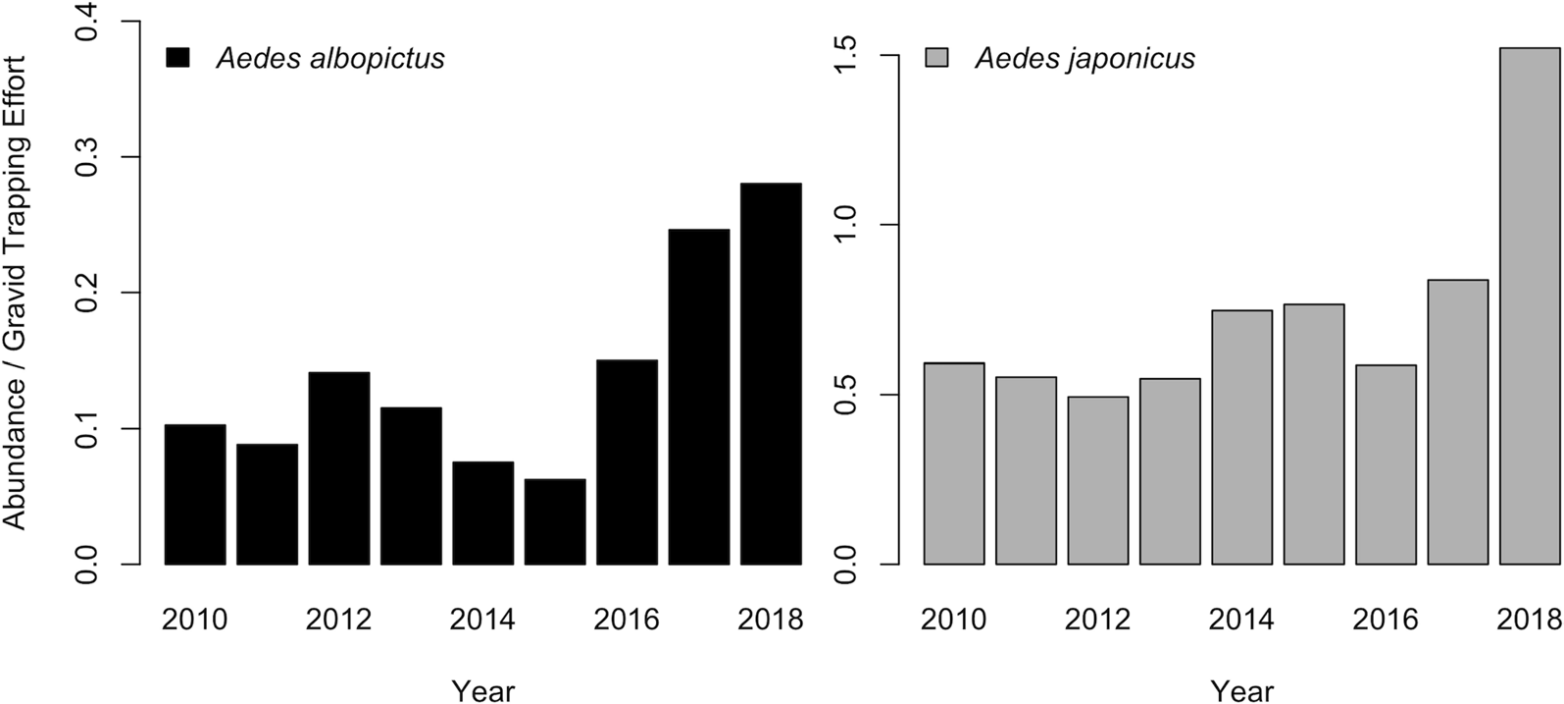
*Aedes albopictus* (left) and *Ae. japonicus* (right) total abundance divided by trap nights across gravid trap sites in Pennsylvania 2010–2018

The abundance of *Ae. albopictus* (odds ratio [OR] = 1.150; 95% confidence interval [CI] = [1.147, 1.154]; *P* < 0.001) and *Ae. japonicus* (OR = 1.124; 95% CI = [1.123, 1.126]; *P* < 0.001) increased between 2010 and 2018 (Fig. 3). We detected *Ae. albopictus* in 39 counties and *Ae. japonicus* in all 67 counties in Pennsylvania (Fig. 1a and b). The models suggest that the spatial extent of *Ae. albopictus* increased (OR = 1.084; 95% CI = [1.03, 1.141]; *P* = 0.002) while the spatial extent of *Ae. japonicus* did not change (OR = 0.993; 95% CI = [0.961, 1.026]; *P* = 0.667) between 2010 and 2018.

### Influence of urban landscape on the abundance of *Ae. albopictus* and *Ae. japonicus*

**Fig. 4:**
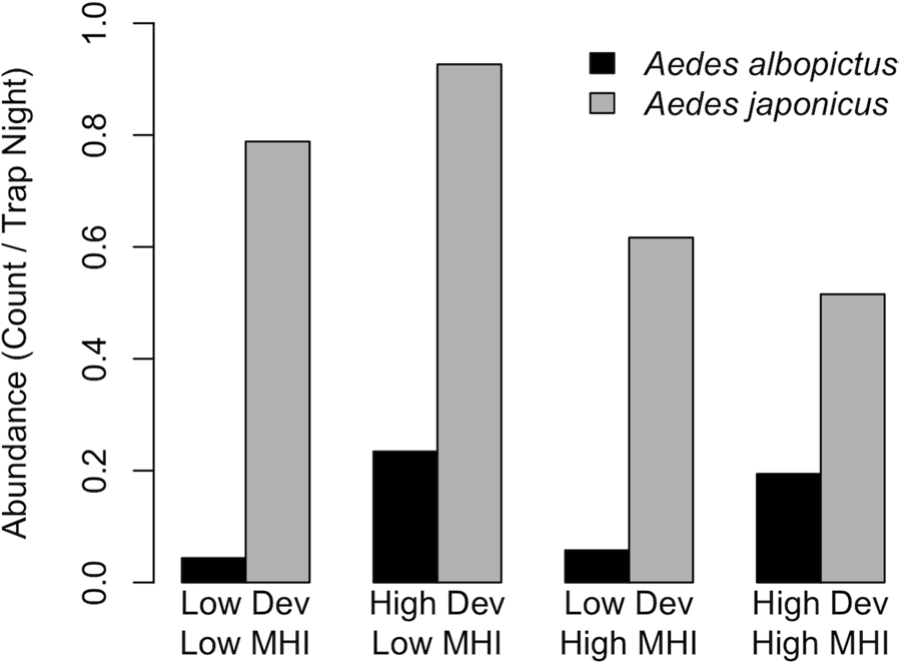
*Aedes albopictus* and *Ae. japonicus* abundance across urban landscapes, percent development and median household income, both stratified at the mean

We found that *Ae. albopictus* and *Ae. japonicus* were associated with environmental conditions, DEV and MHI. *Aedes albopictus* abundance was positively associated with DEV (OR = 2.666; 95% CI = [2.623, 2.704]; *P* < 0.001) and MHI (OR = 1.059; 95% CI = [1.048, 1.070]; *P* < 0.001) (Table 2). The interaction between DEV and MHI was also significant, and areas of higher DEV (above the mean) and lower MHI (below the mean) had the greatest abundance of *Ae. albopictus* (OR = 0.749; 95% CI = [0.741, 0.758]; *P* < 0.001) (Fig. 4). *Aedes japonicus* abundance was negatively associated with DEV (OR = 0.951; 95% CI = [0.948, 0.955]: *P* < 0.001) and MHI (OR = 0.781; 95% CI = [0.777, 0.784]: *P* < 0.001) (Table 2) and was abundant across all urban environments, with the highest abundance in areas with lower MHI (below the mean) compared to other areas (OR = 0.917; 95% CI = [0.912, 0.921]; *P* < 0.001) (Fig. 4).

**Table 2:** Poisson mixed-effect regression model testing the effect of development (DEV), median household income (MHI), and the interaction between development and median household income (DEV × MHI) on *Ae. albopictus* and *Ae. japonicus* collected in gravid traps in Pennsylvania 2010–2018

	*Ae. albopictus*			*Ae. japonicus*		
	OR	95% CI	*P*-value	OR	95% CI	*P*-value
Intercept	0.031	(0.024, 0.039)	< 0.001	0.246	(0.203, 0.298)	< 0.001
DEV	2.666	(2.623, 2.704)	< 0.001	0.951	(0.948, 0.955)	< 0.001
MHI	1.059	(1.048, 1.070)	< 0.001	0.781	(0.777, 0.784)	< 0.001
DEV × MHI	0.749	(0.741, 0.758)	< 0.001	0.917	(0.912, 0.921)	< 0.001

### Blood meal analysis results and influence of urban landscape on blood-feeding patterns

A total of 187 engorged *Ae. albopictus* from 85,824 (0.21%) collected in 2018 were subjected to blood meal analysis. Of these, 58.82% (*n* = 110) had viable results. Most blood meals were identified as a single host 93.64% (*n* = 103). Across single and mixed blood meal results, most included mammalian blood 98.18% (*n* = 108); the three most common hosts were domestic cat, human, and Virginia opossum, representing 43.64% (*n* = 48), 28.18% (*n* = 31), and 13.64% (*n* = 15) of all blood meals analyzed, respectively. Avian blood was identified in 7.27% (*n* = 8) of blood meals analyzed (Table 3). Of the 181,133 *Ae. japonicus* collected between 2010 and 2015, just 97 contained visible blood meals (0.05%). All *Ae. japonicus* fed on mammals, and the most common host was white-tailed deer, representing 79.38% (*n* = 77) of all blood meals analyzed (Table 4).

**Table 3:** Number and percentage of avian- and mammalian-derived blood meals from *Aedes albopictus* collected in Pennsylvania, 2018

Vertebrate hosts Common name (species name)	Frequency of blood meals No. (%)
Mammalian	
Domestic cat (*Felis catus*)	46 (41.82)
Human (*Homo sapiens*)	28 (25.45)
Virginia opossum (*Didelphis virginiana*)	14 (12.73)
White-tailed deer (*Odocoileus virginianus*)	8 (7.27)
Brown rat (*Rattus norvegicus*)	3 (2.73)
Dog (*Canis lupus familiaris*)	1 (0.91)
Red fox (*Vulpes vulpes)*	1 (0.91)
Avian	
House finch (*Haemorhous mexicanus*)	2 (1.82)
Mixed	
Human and house finch (*Homo sapiens* and *Carpodacus mexicanus*)	2 (1.82)
Virginia opossum and house finch (*Didelphis virginiana* and *Carpodacus mexicanus*)	2 (1.82)
Dog and house finch (*Canis lupus familiaris* and *Carpodacus mexicanus*)	1 (0.91)
Domestic cat and house finch (*Felis catus* and *Carpodacus mexicanus*)	1 (0.91)
Domestic cat and human (*Felis catus* and *Homo sapiens*)	1 (0.91)
Total	110 (100)

**Table 4:** Number and percentage of mammalian-derived blood meals from *Aedes japonicus* collected in Pennsylvania, 2010–2015

Vertebrate hosts Common name (species name)	Frequency of blood meals No. (%)
Mammalian	
White-tailed deer (*Odocoileus virginianus*)	77 (79.38)
Domestic cat (*Felis catus*)	6 (6.19)
Dog (*Canis lupus familiaris*)	5 (5.15)
Human (*Homo sapiens*)	4 (4.12)
Eastern cottontail rabbit (*Sylvilagus floridanus*)	2 (2.06)
Cow (*Bos taurus*)	1 (1.03)
Horse (*Equus caballus)*	1 (1.03)
Mixed	
Cat and groundhog (*Felis catus* and *Marmota monax*)	1 (1.03)
Total	97 (100)

To investigate the influence of urban landscapes on *Ae. albopictus* and *Ae. japonicus* blood-feeding, we performed logistic regression with DEV, MHI, and the interaction between these two variables included in the models. We found that *Ae. albopictus* fed more on domestic cats in more highly developed areas with lower MHI and fed more on humans in less developed areas with lower MHI (Table 5). The number of identified blood meals from opossums and white-tailed deer were not sufficient to discern differences in host feeding across urban landscapes. We found that *Ae. japonicus* fed more on white-tailed deer in less developed areas (Table 5).

**Table 5:** Logistic regression results (odds ratios, 95% confidence intervals, and significance level) for *Ae. albopictus* and *Ae. japonicus* blood-feeding

*Ae. albopictus*				
	Domestic cat	Human	Virginia opossum	White-tailed deer
Intercept	0.434 (0.236, 0.745)**	0.525 (0.303, 0.878)*	–	–
DEV	9.864 (2.442, 45.465)**	0.231 (0.079, 0.638)**	–	–
MHI	0.636 (0.290, 1.263)	0.730 (0.319, 1.480)	–	–
DEV × MHI	0.373 (0.181, 0.717)**	11.685 (2.053, 75.505)**	–	–

*Ae. japonicus*				
	White-tailed deer	Domestic cat	Dog	Human
Intercept	4.517 (2.535, 9.291)***	0.032 (0.002, 0.112)***	0.033 (0.004, 0.100)***	0.011 (0.000, 0.078)*
DEV	0.376 (0.155, 0.742)*	5.388 (1.178, 100.273)	2.166 (0.562, 25.398)	10.249 (1.062, 1084.212)
MHI	1.067 (0.547, 2.198)	0.649 (0.158, 2.994)	0.409 (0.104, 1.148)	0.494 (0.076, 3.948)
DEV × MHI	0.596 (0.214, 1.523)	3.351 (0.462, 23.278)	1.362 (0.218, 8.481)	7.267 (0.512, 101.626)

Significance levels: *** refers to a *P*-value of less than 0.001; ** refers to a *P*-value between 0.001 and 0.01; and *refers to a *P*-value between 0.01 and 0.05

### Infection rates of *Ae. albopictus* and *Ae. japonicus*

Between 2010 and 2018, a total of 215,670 pools comprising 9,187,270 mosquitoes across 25 species were tested for WNV, and a subset were tested for LACV and SLEV. The only arbovirus detected was WNV, which was identified in 10 species including in *Ae. albopictus* and *Ae. japonicus* mosquito pools. We calculated the annual MLE for all species that had at least one positive pool (Additional file 1: Table S1). Just 31 of 3582 *Ae. albopictus* and 12 of 977 *Ae. japonicus* pools were positive for WNV. Overall, we found that *Ae. albopictus* had a WNV infection rate of 0.14 (95% CI = [0.10, 0.20]) and *Ae. japonicus* had a WNV infection rate of 0.55 (95% CI = [0.32, 0.96]).

**Fig. 5:**
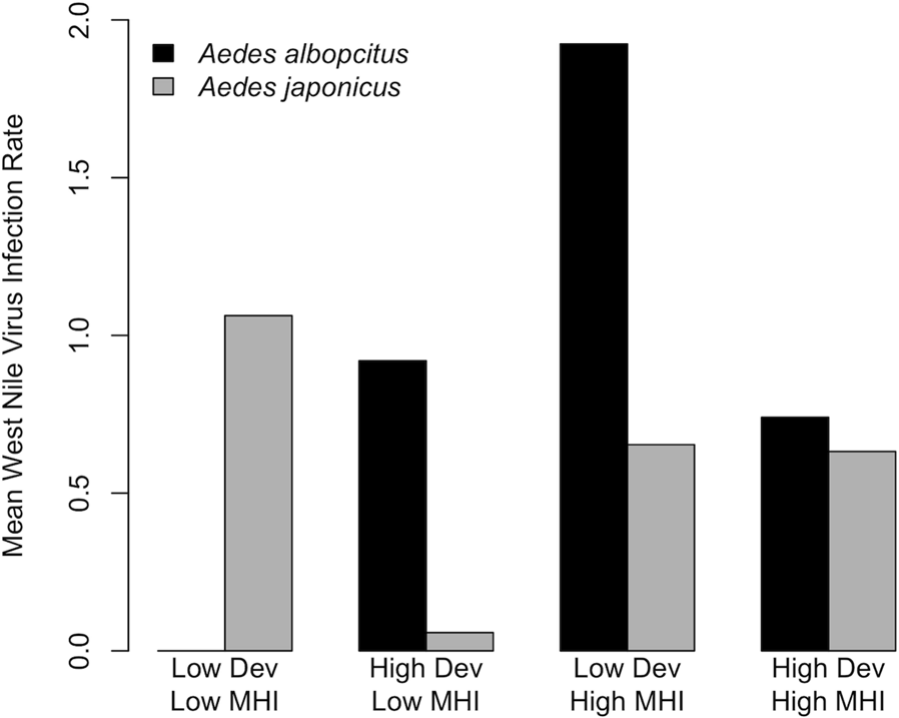
*Aedes albopictus* and *Ae. japonicus* West Nile Virus infection rates across urban landscapes, percent development and median household income, both stratified at the mean

To investigate the influence of urban landscapes on the MLE of WNV infection rates of *Ae. albopictus* and *Ae. japonicus* blood-feeding, we performed generalized linear regression with percent DEV and MHI, and the interaction between these two variables included in the models. *Aedes albopictus* had higher WNV infection rates in areas of lower DEV and higher MHI, while *Ae. japonicus* had higher WNV infection rates in areas of lower DEV and lower MHI compared to other areas (Table 6). While we found a positive association between *Ae. japonicus* WNV infection rates and MHI, the highest infection rates were in areas of low DEV and low MHI (Fig. 5).

**Table 6:** Generalized linear regression model (family = Poisson) testing the effect of development (DEV), median household income (MHI), and the interaction between development and median household income (DEV × MHI) on the maximum likelihood estimation of West Nile virus infection rates of *Ae. albopictus* and *Ae. japonicus* in Pennsylvania, 2010–2018

	*Ae. albopictus*			*Ae. japonicus*		
	OR	95% CI	*P*-value	OR	95% CI	*P*-value
Intercept	0.402	(0.360, 0.447)	< 0.001	0.881	(0.801, 0.966)	0.008
DEV	0.853	(0.775, 0.945)	0.002	0.792	(0.726, 0.865)	< 0.001
MHI	1.543	(1.407, 1.689)	< 0.001	1.189	(1.101, 1.279)	< 0.001
DEV × MHI	1.102	(1.036, 1.176)	0.003	1.253	(1.152, 1.367)	< 0.001

## Discussion

This study provides insight into the distribution, abundance, vector–host interactions, and WNV infection rates of two invasive vectors of arboviruses, *Ae. albopictus* and *Ae. japonicus*, in Pennsylvania. During the study period, 2010–2018, the spatial extent and abundance of *Ae. albopictus* in Pennsylvania increased and the abundance of *Ae. japonicus* also increased. One explanation for the observed increase in the spatial extent of *Ae. albopictus* but not *Ae. japonicus* is that the sampling was conducted largely in urban/suburban habitats, which are more conducive to *Ae. albopictus* than to *Ae. japonicus*. A second possibility is that *Ae. japonicus* has had more time to distribute across the state. As early as 2001, *Ae. japonicus* was common in all 67 counties in Pennsylvania, whereas *Ae. albopictus* was relatively rare.

Identification of greater than 98% of *Ae. albopictus* and 100% of *Ae. japonicus* blood meals acquired from mammalian hosts in this study is in concert with the results of other studies. Studies have shown the percentage of *Ae. albopictus* mammalian-derived blood meals between 71 and 100% [20–32] and between 85 and 100% for *Ae. japonicus* [21, 29, 36–41, 44].

We found frequent interactions of *Ae. albopictus* with humans (27%) and domestic cats (44%) as hosts in our study. While some studies have identified these two mammalian species as the primary hosts for *Ae. albopictus* (between 61 and 100%) [20–27, 30], other studies have reported that 19% and 35% of blood meals for *Ae. albopictus* originated from cottontail rabbits in Missouri [31] and in multiple states (Missouri, Florida, Indiana, Illinois, and Louisiana) [28], respectively. A study in Baltimore, Maryland reported that 72% of blood meals came from rats [29].

Here we found 4% of *Ae. japonicus* blood meals acquired from humans and 79% obtained from white-tailed deer. One study in Belgium found that 60% of *Ae. japonicus *blood meals originated from humans [41]. However, other studies have shown mammals other than humans to be the primary source of blood meal. In Maryland, 50% of blood meals originated from rats [29], and multiple studies have shown that most blood meals (53–100%) were derived from white-tailed deer [36–39]. The frequency of white-tailed deer as hosts for *Ae. japonicus* in these studies is, at least in part, an indication of the abundance of this vertebrate species in these study locations.

Domestic cats in Pennsylvania have not been shown to be infected with arboviruses that infect humans. However, infection of white-tailed deer with WNV, EEEV, LACV, and SLEV in Pennsylvania [80] and with WNV and SLEV in neighboring New Jersey has been reported [81]. White-tailed deer have also been shown to be amplifying hosts of CVV and JCV [82, 83]. In areas with abundant populations of white-tailed deer, they are often targeted by mosquitoes [36]. In Pennsylvania, white-tailed deer support *Ae. japonicus* populations through ample blood meals and have been shown to be infected with arboviruses that can infect humans [80].

Only 7.3% of *Ae. albopictus* obtained blood meals from avian hosts exclusively or in mixed blood meals. Most other studies have also shown birds to be infrequent hosts for *Ae. albopictus* [21, 28, 30, 32, 34]. However, one study in a forested area of China found that avian blood was detected almost as frequently as human blood [84]. Studies in urban areas have also found *Ae. albopictus* to feed on birds; in Missouri, 21% [31] and in Korea, 26% of blood meals were from birds [27]. We did not find any evidence of *Ae. japonicus* avian blood-feeding, which is in accord with most other studies [29, 36–41]. Only one study conducted at an urban zoo in Switzerland found avian blood-feeding. While most *Ae. japonicus* fed on mammals (84.7%), the remaining 15.3% of blood meals originated from birds [44].

In this study we explored the importance of urban development on *Ae. albopictus* and *Ae. japonicus* abundance and blood-feeding. We found greater abundance of *Ae. albopictus* in areas of higher DEV, while more *Ae. japonicus* were found in areas of lower DEV (Table 2). *Aedes albopictus* and *Ae. japonicus* have previously been shown to occupy slightly different niches, with *Ae. albopictus* more abundant in urban and *Ae. japonicus* in rural areas [12]. Adult *Ae. japonicus* show a preference for heavily vegetated areas regardless of the landscape matrix, i.e., agricultural, rural, suburban, or urban [11]. We also found that MHI was significantly related to the abundance of these two mosquito species, with more *Ae. albopictus* found in areas of high MHI and *Ae. japonicus* in areas of low MHI (Table 2). We did find a significant interactive effect between percent DEV and MHI, such that the highest *Ae. japonicus* abundance was in areas of low DEV and low MHI (Table 2; Fig. 4). It is interesting that we found higher *Ae. albopictus* abundance in areas of higher MHI in Pennsylvania while other studies have shown higher *Ae. albopictus* abundance in areas with lower MHI [35, 85]. Differences in the relationship of *Aedes* mosquitoes to DEV and MHI across studies may be driven by variability in container habitat and vegetation across socioeconomic status among other factors [35].

*Aedes albopictus* fed more on domestic cats in more highly developed areas with lower MHI and fed more on humans in less developed areas with lower MHI (Table 5). *Aedes japonicus* fed more on white-tailed deer in less developed areas. Among other factors, these differences in host feeding likely reflect the variation in availability of hosts across urban environments, where blood-feeding frequency can vary by environmental characteristics [30].

The paucity or lack of avian-derived blood meals in field-collected *Ae. albopictus* and *Ae. japonicus* could be due the proximity of the traps to the ground, which may not capture *Ae. albopictus* and *Ae. japonicus* that feed on birds, or simply the difficulty in collecting sufficient number of engorged mosquitoes [29, 38]. In this study, just 0.21% of *Ae. albopictus* and 0.05% of *Ae. japonicus* sampled were blood-engorged. Although low, we did find that 0.14 per 1000 (0.01%) of *Ae. albopictus* and 0.55 per 1000 (0.06%) of *Ae. japonicus* were infected with WNV. Because birds are viewed as the principal reservoir and amplification hosts of WNV, these aforementioned factors may underlie the limitations associated with a relatively small number of engorged mosquitoes analyzed in this study (and by others) and detection of avian-derived blood meals rather than a true absence of avian feeding. Alternatively, it may be possible for *Ae. albopictus* and *Ae. japonicus* to acquire WNV from mammals such as white-tailed deer or eastern chipmunk, as has been suggested by other studies [37, 38].

WNV has been isolated from field-collected *Ae. albopictus* and *Ae. japonicus* in various regions of the United States [45, 61, 62, 86–88]. Isolation of arboviruses including CVV, LACV, JCV, and EEEV has also been reported from wild-caught *Ae. albopictus* [45, 46] and LACV and CVV from *Ae. japonicus* [40, 42, 63] in the United States. The emergence of LACV has been linked to *Ae. albopictus* and *Ae. japonicus* in the Appalachian region of the United States [15]. Human-derived blood meals in concert with the detection of WNV from field-collected *Ae. albopictus* and *Ae. japonicus* in Pennsylvania suggest the potential roles these mosquitoes play as bridge vectors in WNV transmission to humans. More research is needed to investigate titers of WNV and other arboviruses in *Ae. albopictus* and *Ae. japonicus* to determine whether these field-infected mosquitoes can also transmit this arbovirus.

We also investigated whether WNV infection rates in *Ae. albopictus* and *Ae. japonicus* varied with DEV and MHI in Pennsylvania. The WNV infection rate of both species was higher in areas of low DEV. However, *Ae. albopictus* infection rates were higher in areas of high MHI, while *Ae. japonicus* infection rates were higher in areas of low MHI. It is important to note that *Ae. japonicus* WNV infection rates were highest in areas of low DEV and low MHI (Fig. 5). A recent study in Baltimore, Maryland found that WNV infection rates were negatively associated with mean neighborhood income [65]. The limitation of the present study in encountering very few WNV-positive pools of *Ae. albopictus* and *Ae. japonicus* highlights the need for further research in order to draw definitive conclusions about the relationship between these urban characteristics and WNV infection prevalence in these invasive *Aedes* mosquitoes.

## Conclusion

Better understanding of the distribution, abundance, infection prevalence, and host interaction of *Ae. albopictus* and *Ae. japonicus* in nature is vital for assessing their vectorial capacity and contribution to arbovirus transmission in different virus foci. Our study indicates widespread distribution, high abundance, range expansion, and frequent interactions of *Ae. albopictus* and *Ae. japonicus* with mammalian hosts, including humans, and highlights their potential for transmission of arboviruses to humans in the region. Avian-derived blood meals in *Ae. albopictus*, albeit at lower frequency, and infection with arboviruses in field-collected mosquitoes also suggest that this mosquito species might occasionally serve as a bridge vector of WNV to humans and other mammals in the region.

## Supplementary Information

**Additional file 1: Table S1.** The annual infection rate for all species that had at least one positive pool using maximum likelihood estimation (MLE) and 95% confidence intervals in parenthesis.

## Data Availability

Data generated or analyzed during this study are included in this published article and its additional file. Additional data may be available from the corresponding author on reasonable request.
