# Supplementary material for: Spatiotemporal distribution, abundance, and host interactions of two invasive vectors of arboviruses, Aedes albopictus and Aedes japonicus, in Pennsylvania, USA
Source: Parasit Vectors. 2022 Jan 24;15:36. doi: 10.1186/s13071-022-05151-8 (PMC8785538; doi:10.1186/s13071-022-05151-8)
Supplement: Supplementary file 1 — Additional file 1: Table S1. The annual infection rate for all species that had at least one positive pool using maximum likelihood estimation (MLE) and 95% confidence intervals in parenthesis. [file 13071_2022_5151_MOESM1_ESM.docx]

**Supplemental Table 1**. The annual infection rate for all species that had at least one positive pool using maximum likelihood estimation (MLE) and 95% confidence intervals in parenthesis.
